# Comparative malaria impact of chlorfenapyr-α-cypermethrin nets *versus* pyrethroid-only and pyrethroid-piperonyl butoxide nets across high and moderate transmission settings in Tanzania: A modelling study

**DOI:** 10.1016/j.crpvbd.2026.100429

**Published:** 2026-08-25

**Authors:** Gloria Salome Shirima, Silas Steven Mirau, Charles D. Mwalimu, Mponeja Gitanya, Nakul Chitnis, Samson Kiware, Thiery Masserey

**Affiliations:** a Environmental Health and Ecological Science Department, Ifakara Health Institute, P.O. Box 53, Ifakara, Tanzania; b Computational and Communication Science and Engineering, The Nelson Mandela African Institution of Science and Technology (NM-AIST), P.O. Box 447, Tengeru, Arusha, Tanzania; c University of Basel, Petersplatz 1, Basel, 4001, Switzerland; d National Malaria Control Programme (NMCP), Dodoma, Tanzania; e Swiss TPH, Kreuzstrasse 2, Allschwil, 4123, Switzerland; f KCMC University, Kilimanjaro, Tanzania

**Keywords:** Malaria, Insecticide-treated nets, Chlorfenapyr-α-cypermethrin nets, PBO nets, Pyrethroid nets

## Abstract

Malaria persists across sub-Saharan Africa, partly due to insecticide resistance in malaria vectors and the short durability of insecticide-treated nets (ITNs). Next-generation insecticide-treated nets with dual active ingredients (such as chlorfenapyr and alpha-cypermethrin) have been developed to counteract these challenges, but their effectiveness and economic impact compared to current (pyrethroid (PY) and pyrethroid-piperonyl butoxide (PBO)) nets in varied transmission settings remain under evaluation. To inform the Tanzanian National Malaria Control Programme, we used an individual-based model of malaria transmission to assess the epidemiological and economic impacts of three ITN types (chlorfenapyr-α-cypermethrin, PBO, and standard pyrethroid nets). ITN effects were parameterized based on entomological experimental hut trial data in Tanzania. We simulated the deployment of each ITN type at various access levels through three delivery channels (targeted mass campaigns, school net programme, and reproductive and child health) across moderate and high transmission strata representative of Tanzania. We estimated reductions in clinical cases, total costs, and costs per case averted across net types. In both strata, chlorfenapyr-α-cypermethrin nets averted more malaria episodes per person per year for one-round mass distribution compared to PBO and PY nets. The impact remained high through the third-year post-distribution, indicating sustained long-term impact. Despite higher unit costs, chlorfenapyr-α-cypermethrin nets demonstrated a competitive cost per case averted compared to PBO and standard PY nets. These findings provide epidemiological and economic evidence to support Tanzania’s National Malaria Control Programme in optimizing deployments of next-generation nets across delivery channels and transmission strata.

## Highlights

• Chlorfenapyr-α-cypermethrin nets warrant assessment across transmission strata to guide NMCP deployment in Tanzania.
• Chlorfenapyr-α-cypermethrin nets reduced malaria prevalence more than PBO and PY in high and moderate transmission areas.
• Chlorfenapyr-α-cypermethrin nets deliver more sustained epidemiological impact than PBO nets through Year 3.
• Chlorfenapyr-α-cypermethrin nets showed a competitive cost per case averted *versus* PBO and standard pyrethroid nets.

## Graphical abstract

**Figure:**
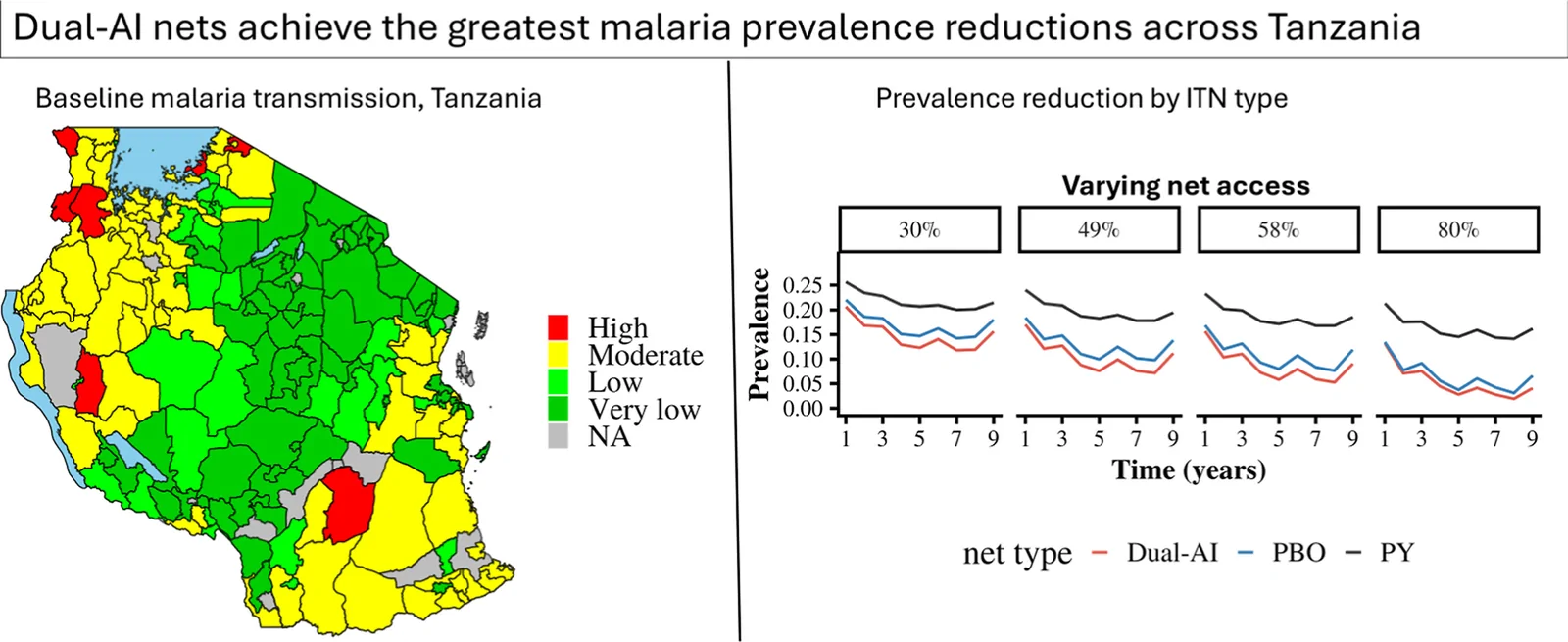

## 1 Introduction

Vector control interventions play a significant role in malaria control, especially in sub-Saharan Africa, where the malaria health burden is high. In these settings, the core vector interventions are insecticide-treated nets (ITN) and indoor residual spraying (IRS) (Bhatt et al., 2015). Initially, nets offered only a physical barrier. The discovery of pyrethroid insecticides led to the development of ITNs that could also kill mosquitoes, leading to an extended protection against malaria. ITNs needed to be retreated regularly. However, the introduction of long-lasting insecticidal nets (LLINs), which have insecticide embedded in the fibers, provided extended protection and longer-lasting efficacy without the need for retreatment (Lengeler, 2004). According to the WHO report, LLINs have contributed to the reduction of malaria case incidence by 50% and reduced mortality among children under 5 years of age by 55% in sub-Saharan Africa (WHO, 2017). However, with increased use of LLINs, mosquitoes evolved resistance against pyrethroids (Keïta et al., 2021; Sanou et al., 2021). This led to the development of pyrethroid-piperonyl butoxide (PBO) ITNs. PBO is a chemical synergist that enhances the pyrethroid, improving its killing effect, particularly against mosquito populations (Hien et al., 2024). However, the effectiveness of PBO nets has been shown to diminish over time, primarily due to the limited durability of PBO on net fibers and the reduced physical longevity of PBO-treated nets compared to standard LLINs (Mosha et al., 2022). Furthermore, in some mosquito populations, PBO only partially restores susceptibility to pyrethroids, and the added value of PBO-based nets appears limited over time, potentially due to durability issues with the synergist and/or reduced textile durability of these nets (Mosha et al., 2022; Oruni et al., 2024). This incomplete restoration of pyrethroid susceptibility has highlighted the need for new insecticides or novel control strategies (WHO, 2022). This includes next-generation nets such as chlorfenapyr-α-cypermethrin nets having dual active ingredients to counteract mosquito resistance mechanisms.

**Fig. 1:**
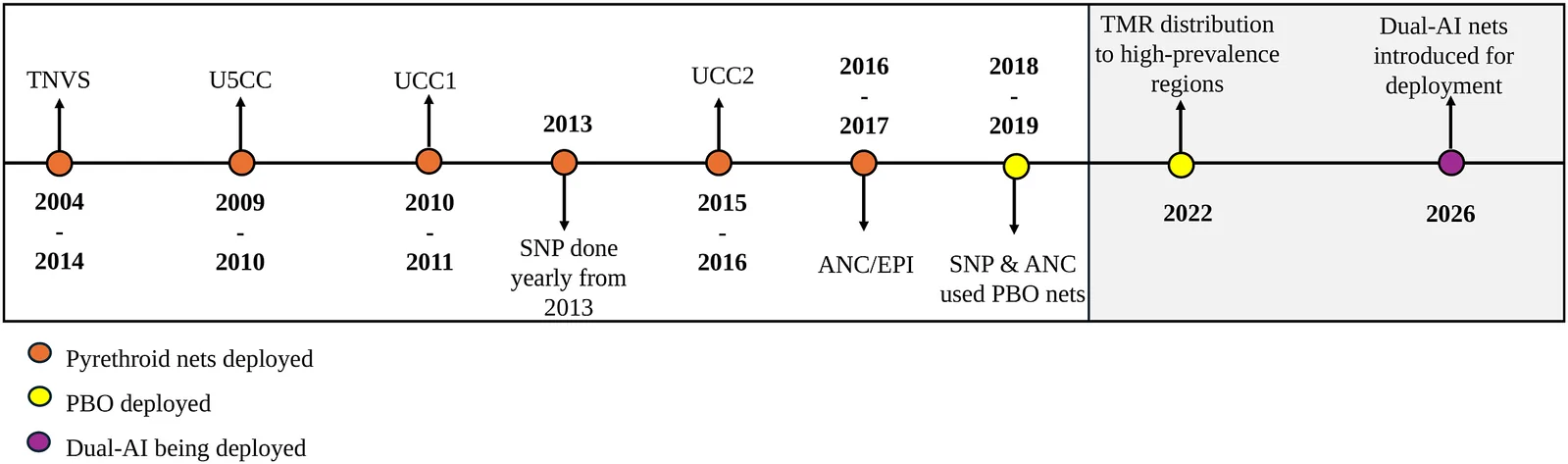
Timeline of long-lasting insecticidal net (LLIN) distribution strategies in mainland Tanzania, 2004–2026. The timeline depicts successive national deployment channels: the Tanzania National Voucher Scheme (TNVS) distributed subsidized nets through reproductive and child health (RCH) facilities; the Under-Five Catch-Up Campaign (U5CC) and two Universal Coverage Campaigns (UCC1 and UCC2) provided free PY nets to achieve universal coverage. The SNP commenced in 2013 with annual distribution through primary schools. From 2016, routine distribution through antenatal care (ANC) and the Expanded Programme on Immunization (EPI) was reinstated at national scale. PBO nets were introduced through SNP and ANC channels from 2018. From 2022, TMR campaigns were implemented in high- and moderate-transmission regions, under the 2021–2025 National Malaria Strategic Plan. TMR is conducted every three years alongside SNP conducted between the TMR campaigns and continuous RCH distribution. The 2026 cycle will introduce dual active ingredients (dual-AI) nets.

Tanzania is one of the sub-Saharan African countries at the forefront of the fight against malaria. The deployment of ITN, IRS, and intermittent preventive treatment has significantly reduced malaria prevalence in Tanzania over the last decades from 18% in 2008 to 5% in 2017 (MoH, 2010, 2017) for children under 5 years of age. In 2014, Tanzania had already established a strong ITN distribution channel across different transmission strata (zones) through a voucher scheme (Kramer et al., 2017; NMCP, 2020). ITNs are currently distributed through both discrete and continuous delivery channels (Fig. 1). The discrete channels include Targeted Mass Campaign (TMC), involving large-scale campaigns conducted approximately every three years to replace worn-out nets, and School Net Programme (SNP), targeting school-aged children through scheduled school distributions between TMCs. A continuous channel is the Reproductive and Child Health (RCH) clinics, which provide nets to pregnant women and caregivers during antenatal and immunization visits (Fig. 1). Together, these approaches sustain ITN coverage and have substantially increased national ITN access. A key question facing the National Malaria Control Programme (NMCP) is which net type should be deployed across these channels to maximize and sustain protection, given the heterogeneous transmission and resistance landscape across Tanzanian councils. However, as of the last deployment, the ITNs distributed are PBO in all channels, and PBO may have limited durability of effects (Protopopoff et al., 2018). The distribution interval for the TMC is typically three years in moderate and high transmission settings in Tanzania, and this interval is longer than the durability of PBO (Mosha et al., 2022). Thus, deploying alternative ITNs that can maintain protection throughout the period between campaigns is essential. Chlorfenapyr-α-cypermethrin ITNs have shown promising results against malaria in areas with highly resistant mosquitoes (Protopopoff et al., 2018) and offer better durability than the PBOs and pyrethroid nets (Champagne et al., 2025). Generating evidence on the optimal deployment of chlorfenapyr-α-cypermethrin nets across Tanzania’s varied transmission strata is therefore a programmatic priority for the NMCP to inform future national procurement and distribution decisions. However, evidence on the performance of chlorfenapyr-α-cypermethrin nets across different transmission strata levels is lacking.

Malaria models have been used to understand disease and transmission dynamics and predict the effectiveness of interventions across various transmission settings to support decisions (Chitnis et al., 2012; Brady et al., 2016; Smith et al., 2018). With transmission dynamics being heterogeneous in Tanzania and the risk of spread of pyrethroid-resistant mosquito populations, modelling is needed to guide the optimal deployment of ITNs through different channels across different transmission levels (Kabula et al., 2011). Here, we used an established individual-based model of malaria transmission, OpenMalaria (Smith et al., 2006; Chitnis et al., 2012), to evaluate the population-level health impact of deploying chlorfenapyr-α-cypermethrin nets through distribution channels including TMC, SNP, and RCH campaigns across moderate- and high-transmission settings, and compared this impact to the deployment of PBO nets and standard pyrethroid-only nets. Based on entomological data, we hypothesized that chlorfenapyr-α-cypermethrin nets offer greater and more sustained protection given their dual mechanism of action and demonstrated activity against pyrethroid-resistant vectors. Then, we examined the time of continuous net deployment needed for each net type to shift a population from moderate or high to low or very low malaria transmission, providing realistic programme timelines and the conditions under which transmission reduction targets are achievable. Finally, we conducted a cost-benefit analysis of each net type at the council level, quantifying the economic value of averted cases, relative to procurement and distribution costs, to inform resource allocation decisions by the national malaria control programme. Together, these analyses aim to support the optimal, evidence-based deployment of ITNs and ensure sustainable, cost-effective malaria control across moderate- and high-transmission settings in sub-Saharan Africa.

## 2 Materials and methods

### 2.1 Model structure

We employed the OpenMalaria model, which integrates an individual-based approach for simulating malaria transmission among humans with a deterministic model for mosquito populations describing their feeding cycle and infectious status. The model has been described extensively previously. Briefly, the human model (Smith et al., 2006, 2008) operates on a 5-day time step, updating outcomes related to malaria cases, the acquisition of immunity, severe cases, and mortality. The mosquito model (Chitnis et al., 2012) operates on a daily time step, updating infection and survival of mosquitoes and subsequent infection of humans. This framework enables the assessment of various malaria interventions across multiple transmission settings with various mosquito species. The model accommodates the specific entomological modes of action associated with different types of nets, such as their mortality effects on mosquitoes before and after blood-feeding, as well as the decay of these effects over time.

### 2.2 Transmission strata and access scenarios

**Fig. 2:**
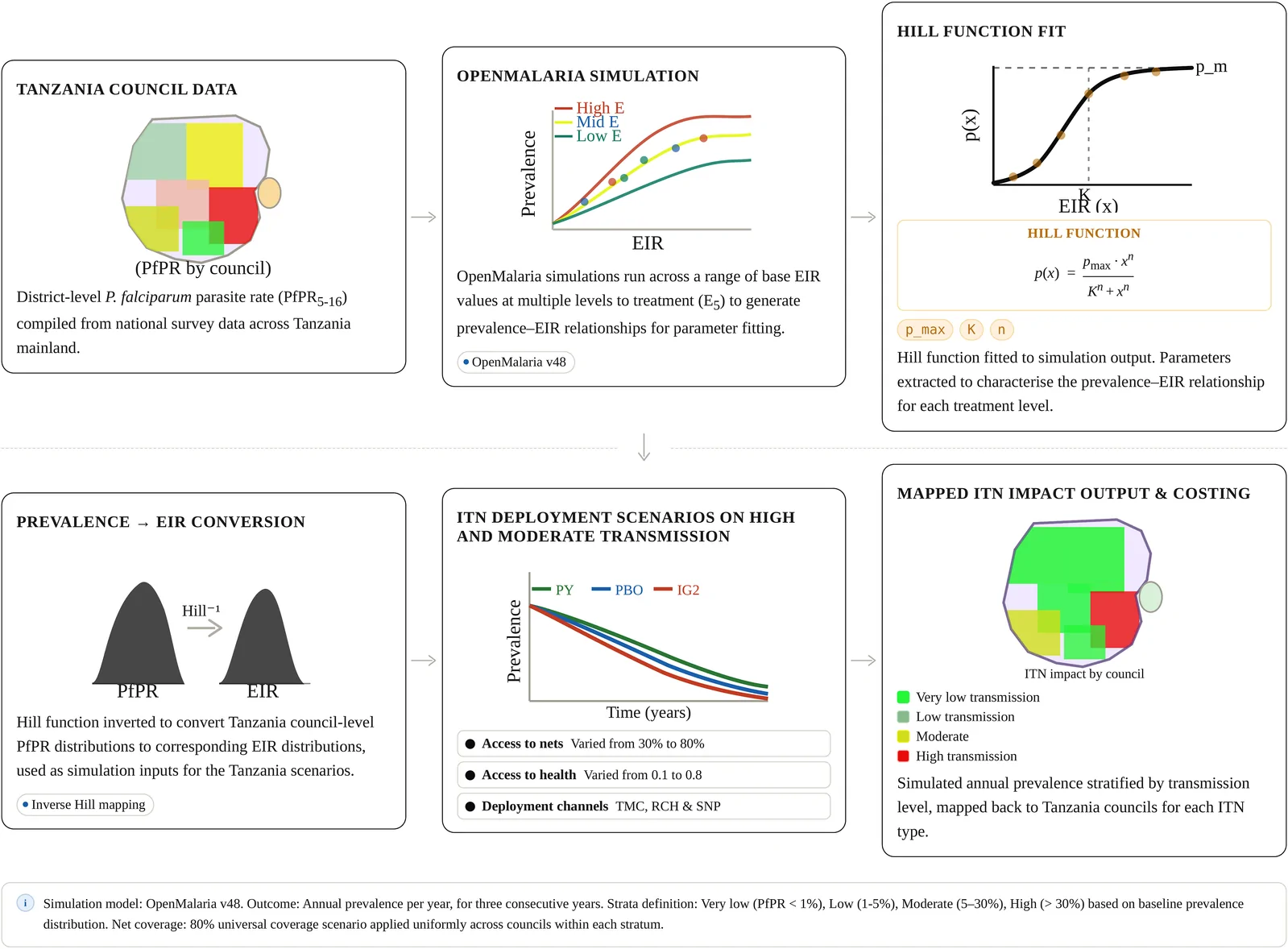
Analytical framework for estimating the epidemiological impact of ITNs types on malaria transmission across Tanzania councils. District-level PfPR_5-16_ data were used to derive council-level EIR *via* inverse Hill function mapping, which served as inputs for OpenMalaria v.48 simulations. Three net types were evaluated across high- and moderate-transmission settings, with simulated annual prevalence mapped back to Tanzania councils alongside cost estimates.

OpenMalaria allows inputs of entomological inoculation rate (EIR) of the setting. Due to the scarcity of EIR data for the councils of interest in Tanzania, we used prevalence data in children aged 5–16 years old (PfPR_5-16_) from the nationwide School Malaria Parasitemia Survey (ages 5–16 years), which provides council-level prevalence at low cost and shows the geographical pattern (Chacky et al., 2018), to design the baseline transmission settings. Prevalence data were converted into EIR using an inverse hill function (Penny et al., 2015) (Supplementary Text S1) (Fig. 2). Note that this relationship depended on the level of access to treatment; thus, we established this relationship across various levels of treatment (10–80%) (Supplementary Fig. S1). The converted EIR values were then used as an input to the model (Fig. 2) to better represent the level of malaria transmission in settings of interest.

To reflect the heterogeneous transmission dynamics, we used the converted EIR as our pre-intervention EIR and varied the access to treatment (10–80%) across simulations. This range was informed by epidemiological data on malaria and healthcare access from Tanzania (Penny et al., 2015; NMCP, 2020; President’s Malaria Initiative, 2023). Classification of transmission levels was based on prevalence levels as stated in the NMSP 2021–2025 (NMCP, 2020). Moderate transmission level corresponded to a prevalence of 5–30%, equivalent (after the Hill function conversion) to EIRs of 2.67–15.27 inoculations per person per year. High transmission level corresponded to prevalence above 30%, which is equivalent to EIR above 15.27 inoculations per person per year.

Note that although we use prevalence among 5–16 year-olds to calibrate the baseline EIR for each council, OpenMalaria can simulate malaria transmission, level of immunity, and disease incidence across the entire population. This is because the core model component of OpenMalaria has been previously parameterized to a wide range of field data to explicitly capture and simulate the relationships between EIR, immunity, malaria incidence, and prevalence across different age groups.

### 2.3 Intervention parameterization and deployment

We evaluated the efficacy of chlorfenapyr-α-cypermethrin nets and PBO nets compared to standard pyrethroid nets across different transmission strata. The different ITNs were parameterized based on experimental hut trials conducted on PY-resistant mosquitoes in Bagamoyo, Tanzania (Massue et al., 2019). These trials provided critical parameters related to mosquito survival, feeding behavior, and the effectiveness of these nets. A previous modelling study (Martin et al., 2024; Champagne et al., 2025) parameterized the pre-prandial killing effect, post-prandial killing effect and deterrence for pyrethroid, PBO, and chlorfenapyr-α-cypermethrin nets to these experimental hut data in OpenMalaria. We used these parameterizations in our study (Supplementary Table S1).

**Fig. 3:**
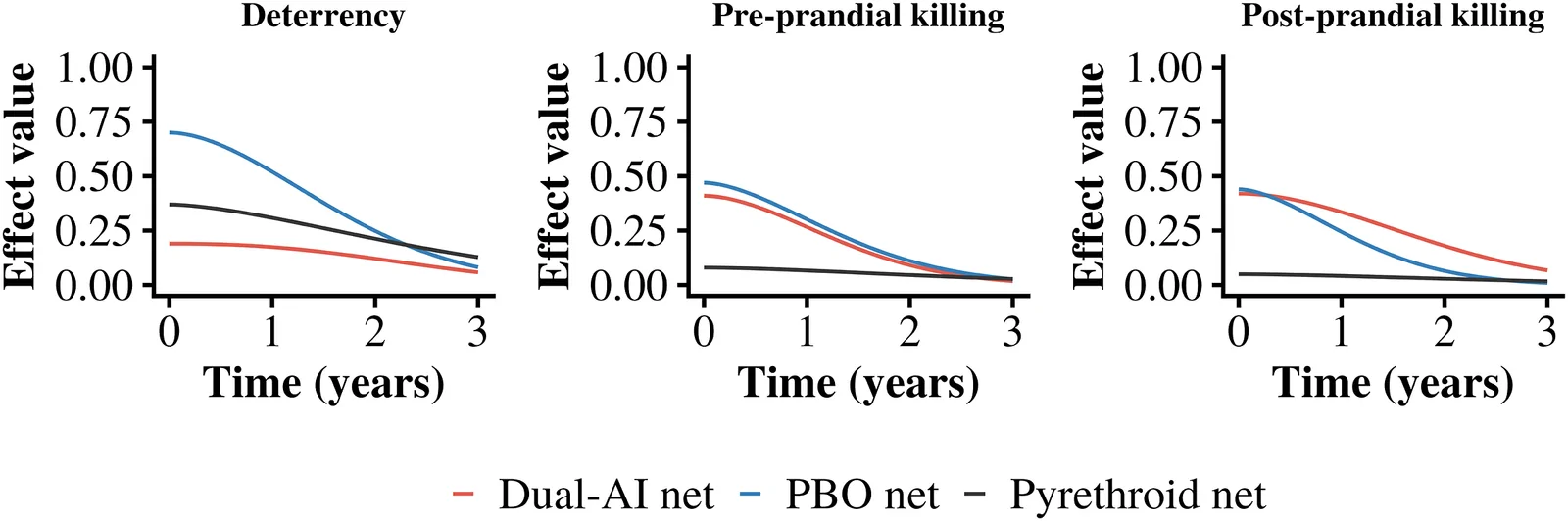
Weibull decay of ITN vector effects over time across three panels: deterrence, pre-prandial killing, and post-prandial killing. Initial effect values reflect net-type-specific efficacy against pyrethroid-resistant *Anopheles* spp. mosquitoes in Tanzania, derived from field and experimental hut studies. PBO nets show the highest initial deterrence, while chlorfenapyr-α-cypermethrin dual-AI net and PBO nets exhibit comparable and substantially higher initial killing effects than pyrethroid-only nets. The decay in effect values over time reflects two compounding processes: gradual degradation of insecticidal activity on the net fabric; and progressive household net loss due to physical attrition and reduced retention. The slower decay of chlorfenapyr-α-cypermethrin net post-prandial killing effects relative to PBO nets underpins the more sustained epidemiological impact observed in the simulations.

In addition, we modelled the effect of each ITN decay over time (Fig. 3) as estimated in Champagne et al. (2025) and Martin et al. (2024) using a Weibull function and adapting decay rate parameters K and L, as shown in Supplementary Table S2 for each net type. This decay captures two distinct processes: (i) insecticidal decay - the physical degradation of insecticidal efficacy over time (lethality), informed by experimental hut trials on progressively washed nets; and (ii) usage decay - the declining rate of net use over time among recipients, informed by clinical trial data from Tanzania (Martin et al., 2024).

We modelled three intervention scenarios, each deploying a different ITN type, i.e. PY nets (baseline), PBO nets, or chlorfenapyr-α-cypermethrin nets, across the same three distribution channels: TMC, RCH, and SNP. The three distribution channels operated as follows: (i) TMC: Mass distribution campaigns targeting the entire population regardless of age, occurring every three years on a discrete schedule. Access to nets for TMC was varied (30–80%) to simulate different numbers of people with ITNs; (ii) RCH: Continuous distribution targeting young children (infants), through routine health contact points, reaching approximately 4% of the total population annually (President’s Malaria Initiative, 2019), with nets provided to all eligible children (infants) who access these services; (iii) SNP: Targeted distribution to school-aged children (5–16 years) covering 3% of that population. Note that school-aged children represent more than 3% of the population, but the 3% coverage was calculated based on the residual gap needed to maintain 80% population access after RCH distribution (i.e. approximately once per year in the non-TMC years over the three-year cycle), and this varies per cycle.

### 2.4 Simulation

Each simulation was performed using ten stochastic seeds to capture variability in outcomes. Each simulation began with a “warm-up” period to establish a stable endemic state prior to the implementation of interventions. Then, the ITNs were deployed according to the scenario (Baseline Scenario, PBO Scenario, and Chlorfenapyr-α-cypermethrin Scenario). We monitored the number of uncomplicated clinical malaria cases in four age groups, those under 1 year, 1–5 years, 5–16 years and above 16 years of age for three ITN deployment cycles (9 years). Cases were also summed across all age groups to analyze the impact of the scenarios on the number of uncomplicated cases (human morbidity) in the whole population. The effectiveness of the PBO Scenario and Chlorfenapyr-α-cypermethrin Scenario were evaluated by measuring the relative reduction in clinical malaria cases, prevalence and episodes compared to the Baseline Scenario (pyrethroid nets).

### 2.5 Cost-benefit analysis

To evaluate the cost-effectiveness of each net type, a cost-benefit analysis was conducted from a programmatic provider perspective, considering consumable procurement costs and distribution costs. While the distribution cost differs per distribution channel, this cost involves human resources, training, and transportation costs. Since OpenMalaria does not generate cost output directly, deployment costs for nets in each council were matched to the number of nets deployed, and cases averted in that council over a 3-year distribution cycle, obtained from the simulation to calculate the cost per case averted. Costs and cases averted were accumulated over a full three-year distribution cycle, including one TMC campaign, two SNP distributions, and continuous RCH delivery to reflect the complete programmatic planning period used in the simulations. Three delivery channels were modelled reflecting Tanzania’s national ITN system: the TMC, assuming one net per two persons approximately distributed once per cycle; the RCH channel, targeting infants at nine months of age (estimated as 4% of the population annually); and the SNP, targeting children aged 5–16 years (estimated at 3% of that population) with one net per child per year. Unit procurement costs differed by net type: the unit cost per net used in the analysis is 3.76 USD for chlorfenapyr and α-cypermethrin, 2.97 USD for PBO and 2.05 USD for PY. Total costs for deploying PY nets, including distribution and unit cost per person, are 1.678 USD for TMC, 2.993 USD for RCH, and 3.01 USD for SNP. For PBO, these values are 2.19 USD for TMC, 3.913 USD for RCH, and 3.93 USD for SNP. Lastly, total costs for chlorfenapyr-α-cypermethrin are 2.628 USD for TMC, 3.937 USD for RCH and 4.720 USD for SNP. The cost for TMC is seen to be lower, as one net is calculated for 1.8 people in the population.

Total three-year deployment cost per 10,000 population was calculated as:

Total cost = (TMC nets × total_cost_TMC) + (RCH nets_3yr × total_cost_RCH) + (SNP nets_3yr × total_cost_SNP),

where TMC nets (number of ITNs deployed through TMC) = (population × 0.80)/2, RCH nets over three years (number of ITNs deployed through RCH) = population × 0.04 × 3, and SNP nets over three years (number of ITNs deployed through SNP) = population × 0.03 × 2. The primary cost-benefit metric was cost per case averted (USD/case averted), calculated by dividing the total 3-year deployment cost by cumulative cases averted over the same period as estimated by OpenMalaria for each council, relative to the pyrethroid-only counterfactual baseline. All calculations were implemented in R and are interactively accessible across all 78 simulated councils *via* the R *Shiny* application (https://doi.org/10.5281/zenodo.20209778).

## 3 Results

### 3.1 Overview of simulated councils and transmission settings

**Fig. 4:**
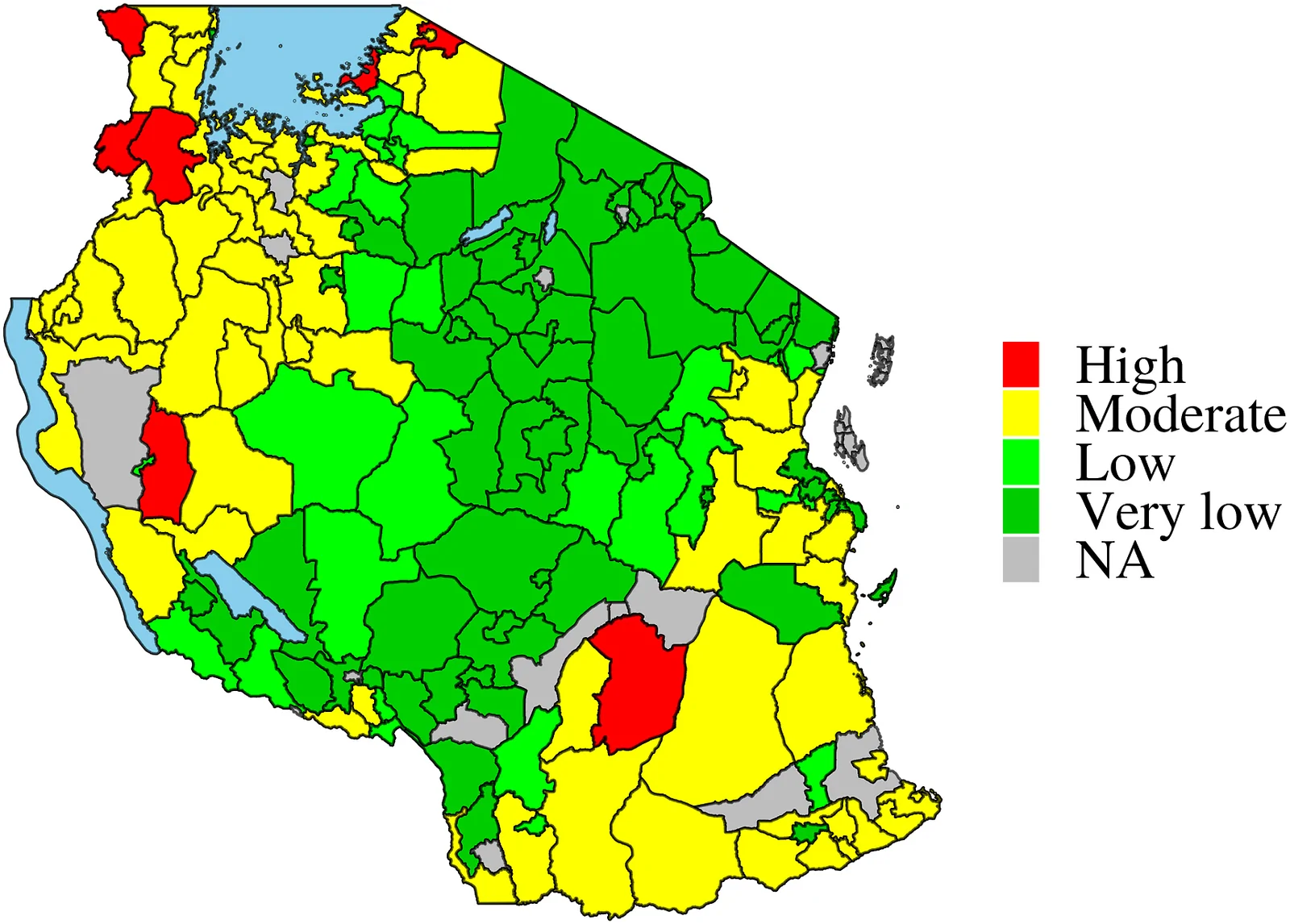
Council-level malaria transmission strata across Tanzania mainland based on *Plasmodium falciparum* parasite rate (PfPR_5-16_) before simulation of net deployments. Councils are classified into four transmission strata: high (*red*), moderate (*yellow*), low (*light green*), and very low (*dark green*); NA indicates the councils with no data (*grey*). Stratification was derived from national survey prevalence data and used as inputs for OpenMalaria simulations when converted to EIR.

Simulations were conducted across 78 councils (out of the 184 councils) that span a range of moderate and high transmission intensities before deployment of interventions. Councils were classified into four broad transmission categories: high (prevalence > 30%); moderate (prevalence of 5–30%); low (prevalence of 1–5%); and very low (prevalence < 1%) (Fig. 4). No deployment of nets is assumed to occur in councils with low and very low transmission. The geographical distribution of these transmission categories across Tanzania is shown in Fig. 4, illustrating substantial heterogeneity in burden at the council level and indicating councils that are potential for TMC deployment.

Given the large number of councils and the multiple combinations of net type, net access level, and healthcare access modelled, an interactive R *Shiny* application was developed to enable flexible visualization of simulation outputs at the council level (https://doi.org/10.5281/zenodo.20209778). The application allows users to explore prevalence trajectories, cumulative cases averted, year-specific impacts across access strata, and cost-benefit estimates for each council and net type combination. For the purposes of this paper, the Mara region was selected as a case study to illustrate findings in depth. Mara was chosen because it contains councils operating under both moderate and high transmission levels before simulation of net deployment, making it representative of the heterogeneity observed across Tanzania and well-suited to addressing all three study aims.

### 3.2 Impact on malaria morbidity: Mara region

#### 3.2.1 Comparative effectiveness across net types

**Fig. 5:**
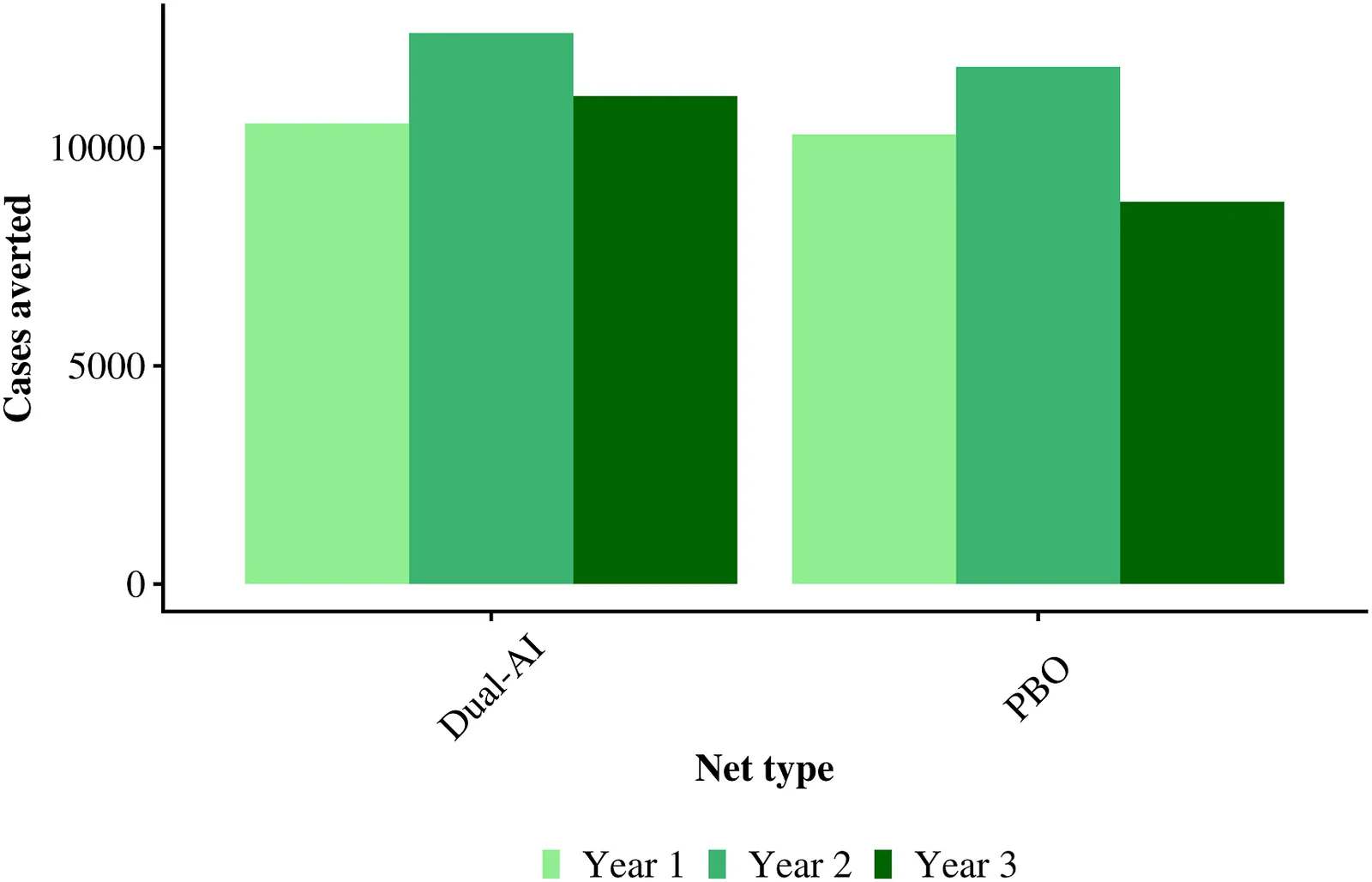
Malaria cases averted by net type over 3 years of deployment. Comparison of cases averted following deployment of chlorfenapyr and α-cypermethrin nets (Dual-AI) versus PBO nets across Years 1, 2, and 3. These are additional cases averted in comparison to PY nets as baseline. Dual-AI nets demonstrated sustained case aversion across the study period, while PBO nets showed a decline in cases averted by Year 3, suggesting differential effectiveness and durability between net types. *Abbreviations*: Dual-AI, dual active ingredient; PBO, piperonyl butoxide. PY, pyrethroid-only.

In all Mara councils, all intervention scenarios of chlorfenapyr-α-cypermethrin and PBO substantially reduced uncomplicated malaria cases compared with the baseline pyrethroid-only ITN scenario. Over the first three years following net distribution in a simulated population of 10,000 individuals, chlorfenapyr-α-cypermethrin nets averted a total of 34,366 cases relative to the pyrethroid-only baseline, while PBO nets averted 30,916 cases over the same period at 80% net access and 0.6 access to treatment. When compared directly, chlorfenapyr-α-cypermethrin achieved a 1.11-fold (95% CI: 0.76–1.63) greater cumulative reduction in uncomplicated malaria cases relative to PBO (Fig. 5). This advantage was consistent across both moderate and high transmission councils within the Mara region and was maintained across all net access levels examined (can be seen from the R *Shiny* tool). Year-specific results demonstrate meaningful temporal variation in intervention impact across both net types (Fig. 5). In the first year following distribution, chlorfenapyr-α-cypermethrin nets averted 10,557 cases compared with 10,303 cases averted by PBO nets. The second year showed the largest absolute reductions across both scenarios: chlorfenapyr-α-cypermethrin averted 12,629 cases, and PBO averted 11,854 cases, reflecting the compounding effect of reduced transmission pressure as the protected population grows. By the third year, impact declined for both net types as a result of net attrition and waning insecticide activity; however, the reduction remained substantially greater for chlorfenapyr-α-cypermethrin (11,181 cases) than for PBO (8757 cases), as chlorfenapyr-α-cypermethrin nets maintain a more durable protective advantage over time in our parameterizations, all at 80% net access and 0.6 access to treatment condition. These comparisons of the cases averted are additional cases that PBO and chlorfenapyr-α-cypermethrin have averted over the baseline PY nets.

**Fig. 6:**
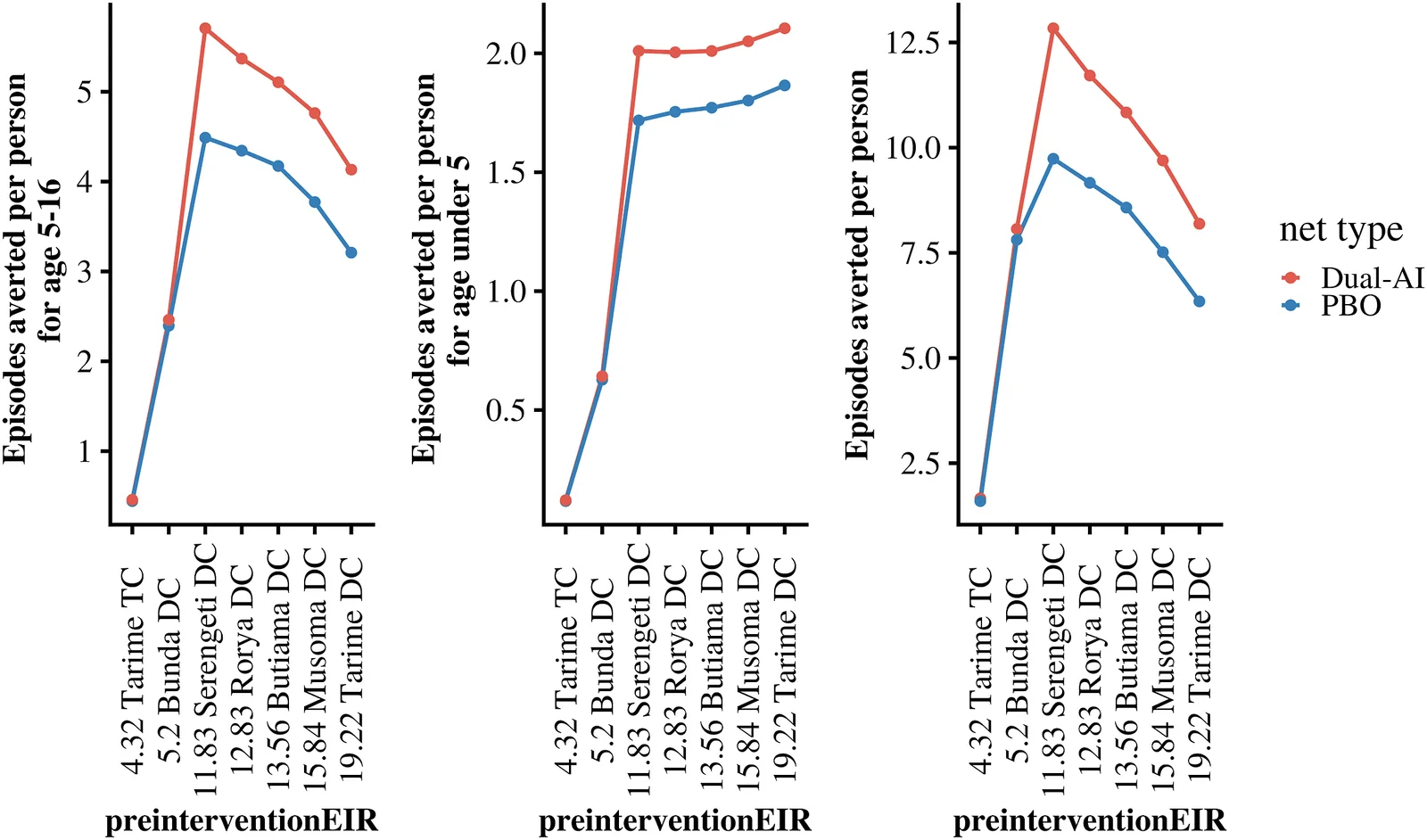
Episodes averted per person over the 3-year deployment of chlorfenapyr-α-cypermethrin and PBO strategies across transmission intensities, stratified by age group. *Abbreviations*: Dual-AI, dual active ingredient; PBO, piperonyl butoxide.

While PBO achieved comparable or marginally lower incidence than chlorfenapyr-α-cypermethrin in specific age groups (Fig. 6) particularly in a few low-transmission councils in Mara (Tarime town council (TC) and Bunda district council (DC)), this advantage was limited and observed in only a small number of councils. However, when examining cumulative cases averted across all age groups, survey rounds, and years, chlorfenapyr-α-cypermethrin consistently outperformed PBO even in these councils. In all other councils, the dual-AI nets demonstrated superior performance across all age groups. The cumulative measure captures the full effect of each intervention, including downstream reductions in transmission that single timepoint comparisons do not reveal.

#### 3.2.2 Temporal patterns of impact

**Fig. 7:**
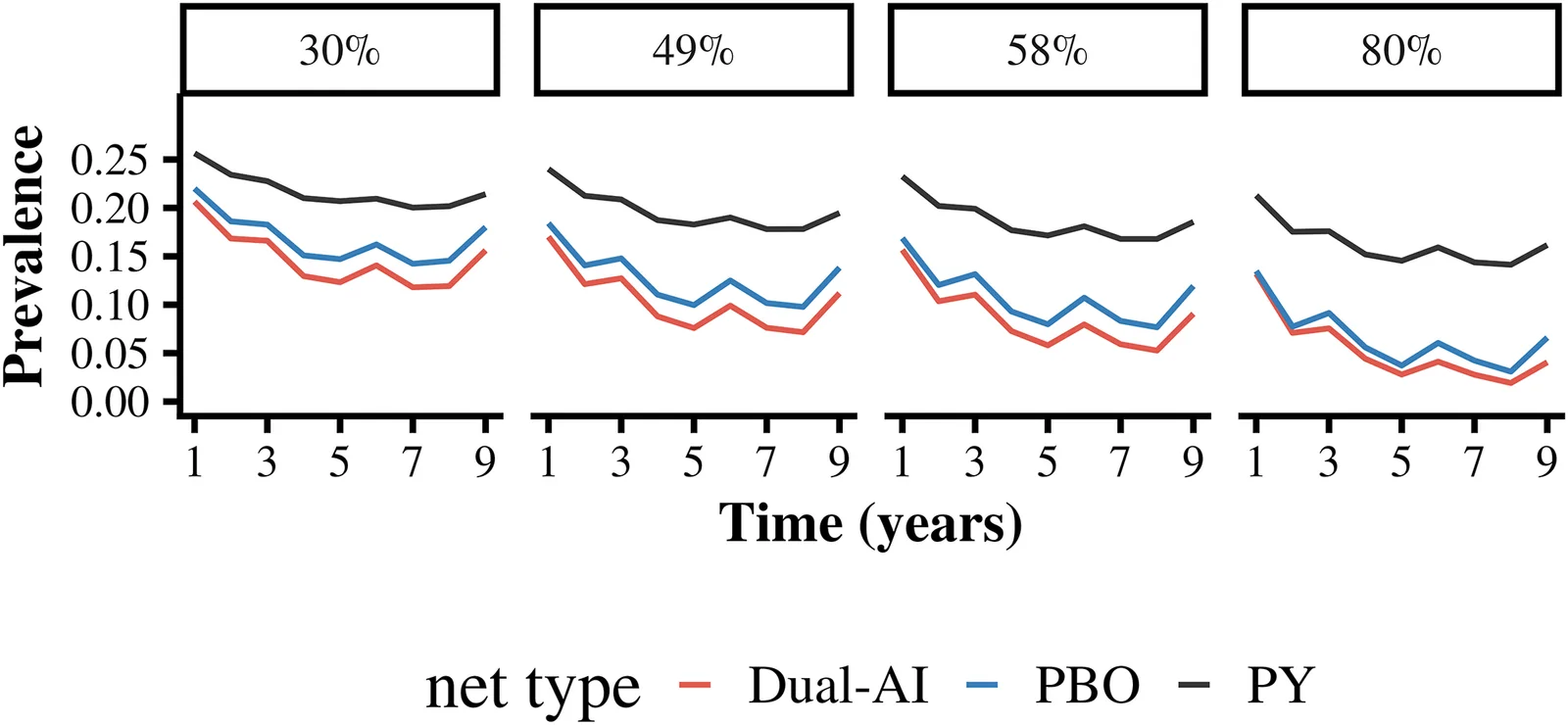
Malaria prevalence over nine years of simulation in Mara region by net type (chlorfenapyr and α-cypermethrin, PBO, PY) across four levels of net access. Each panel represents one access to nets stratum. *Abbreviations*: Dual-AI, dual active ingredient; PBO, piperonyl butoxide. PY, pyrethroid-only.

**Fig. 8:**
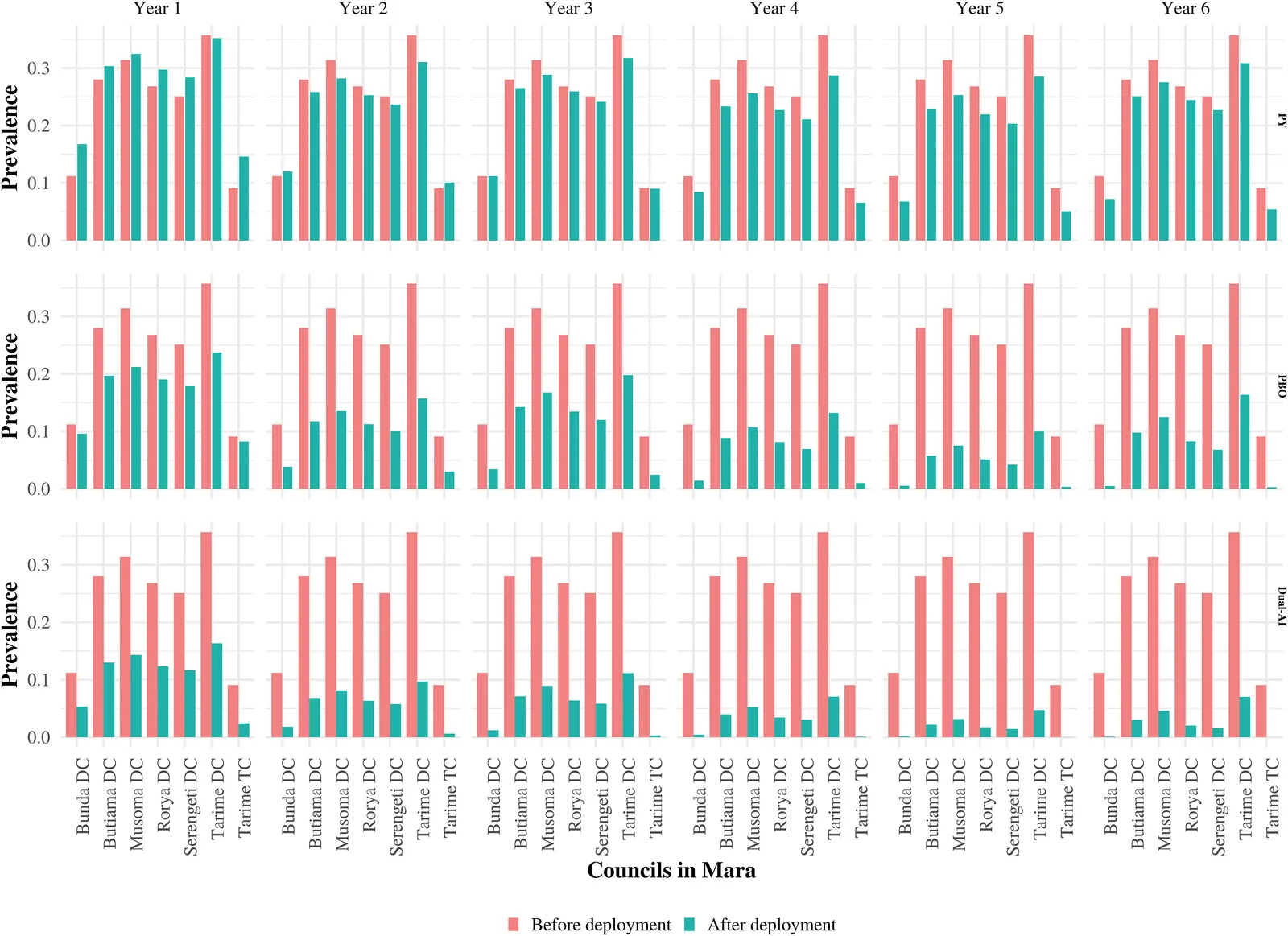
Malaria prevalence by council across 6 years at 80% and 0.6 access. Red bars indicate prevalence before intervention, while teal bars show prevalence after LLIN deployment, demonstrating variable prevalence changes across councils and net types.

Prevalence trajectories across varying access to nets levels (30%, 49%, 58%, and 80%) over nine years of simulation for each net type are shown in Fig. 7. Across all years and access to nets levels, increasing net access from 30% to 80% produced a progressively greater reduction in prevalence for chlorfenapyr-α-cypermethrin and PBO relative to the pyrethroid-only baseline. The impact shown in Fig. 7 is simulated at 0.6 access to treatment. This reduction seen is compared to the transmission level before deployment of nets in each council, and it reduces more over the years of continuous deployment of nets, according to the channels in the strategic plan. As shown in Fig. 8, the magnitude of incidence reduction varies across councils depending on net type deployed, where chlorfenapyr-α-cypermethrin nets lead in the reduction of incidence over the years.

#### 3.2.3 Time to transmission reduction

**Fig. 9:**
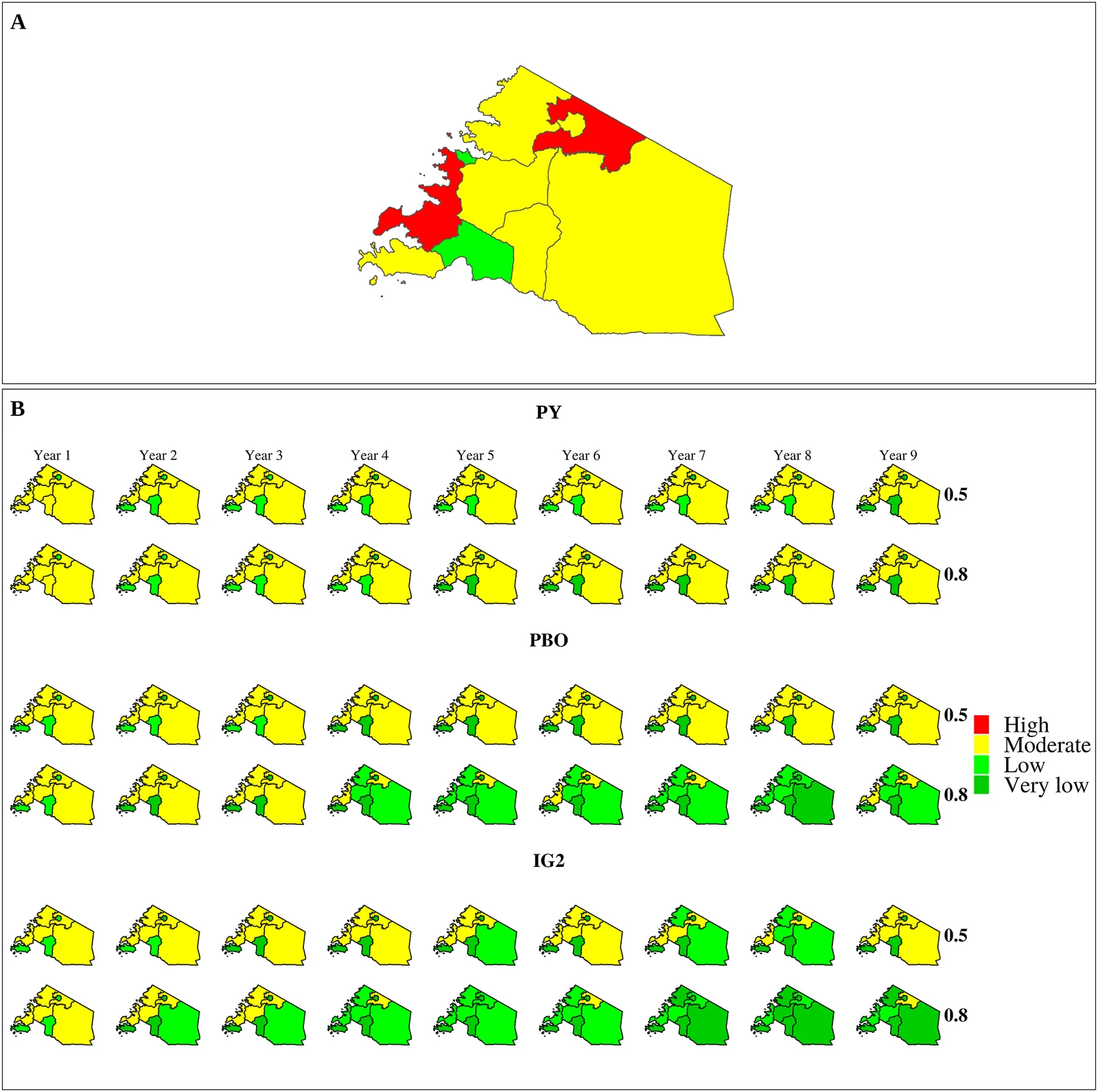
**A** Transmission classification of Mara region councils prior to net deployment, defined by parasite prevalence according to NMCP thresholds: high (> 30%, *red*), moderate (5–30%, *yellow*), low (1–5%, *light green*). **B** Year-by-year transmission stratum classification across Mara councils for chlorfenapyr-α-cypermethrin (Dual-AI), pyrethroid-piperonyl butoxide (PBO), and pyrethroid-only (PY) nets over nine years of deployment, shown at 50% (top row of each panel) and 80% (bottom row of each panel) net access. Color coding follows NMCP thresholds on transmission level: high (*red*), moderate (*yellow*), low (*light green*), very low (*dark green*). Each map panel represents one year of simulation for a given net type and net access level.

Transmission intensity was classified according to National Malaria Control Programme (NMCP) thresholds based on parasite prevalence: very low (< 1%), low (1–5%), moderate (5–30%), and high (> 30%). Classification in this study relied exclusively on modelled prevalence estimates to assign each council to a transmission stratum at each timepoint, consistent with this framework. The transmission profile of the Mara region before simulating the net deployment is shown in Fig. 9A. At the start of the simulation period, prior to any net deployment, two councils were classified as high transmission settings (prevalence > 30%), while the remaining councils fell within the moderate transmission range (prevalence 5–30%). Two councils in Mara region had low baseline transmission and did not receive net deployment, so they were excluded from the intervention scenarios. Of the remaining seven councils, two councils had high transmission, and five councils had moderate transmission. This baseline heterogeneity establishes a starting condition in which sustained intervention would be necessary to achieve meaningful downward transmission.

A clear and consistent pattern emerges, i.e. higher net access produces faster and more complete downward transitions across all net types (Fig. 9B). At 80% net access, chlorfenapyr-α-cypermethrin nets achieved full regional classification within the low or very low transmission stratum by the second deployment cycle, with all Mara councils sustaining this classification through the remainder of the simulation period. This represents a marked acceleration relative to lower net access scenarios and reflects the dual mechanism of action of chlorfenapyr-α-cypermethrin, which retains efficacy against pyrethroid-resistant vectors that would otherwise limit the impact of conventional nets. Under PBO nets at 80% net access, the majority of councils transitioned to low transmission over the simulation period; however, councils that were initially classified as high transmission took longer to descend below the moderate threshold and, in some years between deployment rounds, briefly returned to moderate classification. This rebound between distribution cycles visible in the interannual variation in Fig. 9B highlights the importance of deployment timing and net access maintenance in sustaining transmission gains. PBO nets nonetheless substantially outperformed pyrethroid-only nets, under which the two high-transmission councils pre-net deployment showed minimal or delayed stratum transition even at 80% net access, consistent with the documented activity of pyrethroid-resistant vector populations in the region. At 50% net access, the pace of stratum transition was slower for all net types. Chlorfenapyr-α-cypermethrin nets still produced progressive reductions across councils, but the transition to low or very low transmission was not fully consolidated until later in the simulation horizon. PBO and PY nets at 50% net access showed more limited progress, with several councils remaining in the moderate stratum throughout much of the 9-year period. Full council-level time-to-transition results across all 78 simulated councils are available in the interactive R *Shiny* application (https://doi.org/10.5281/zenodo.20209778).

#### 3.2.4 Cost-benefit analysis at the council level

**Fig. 10:**
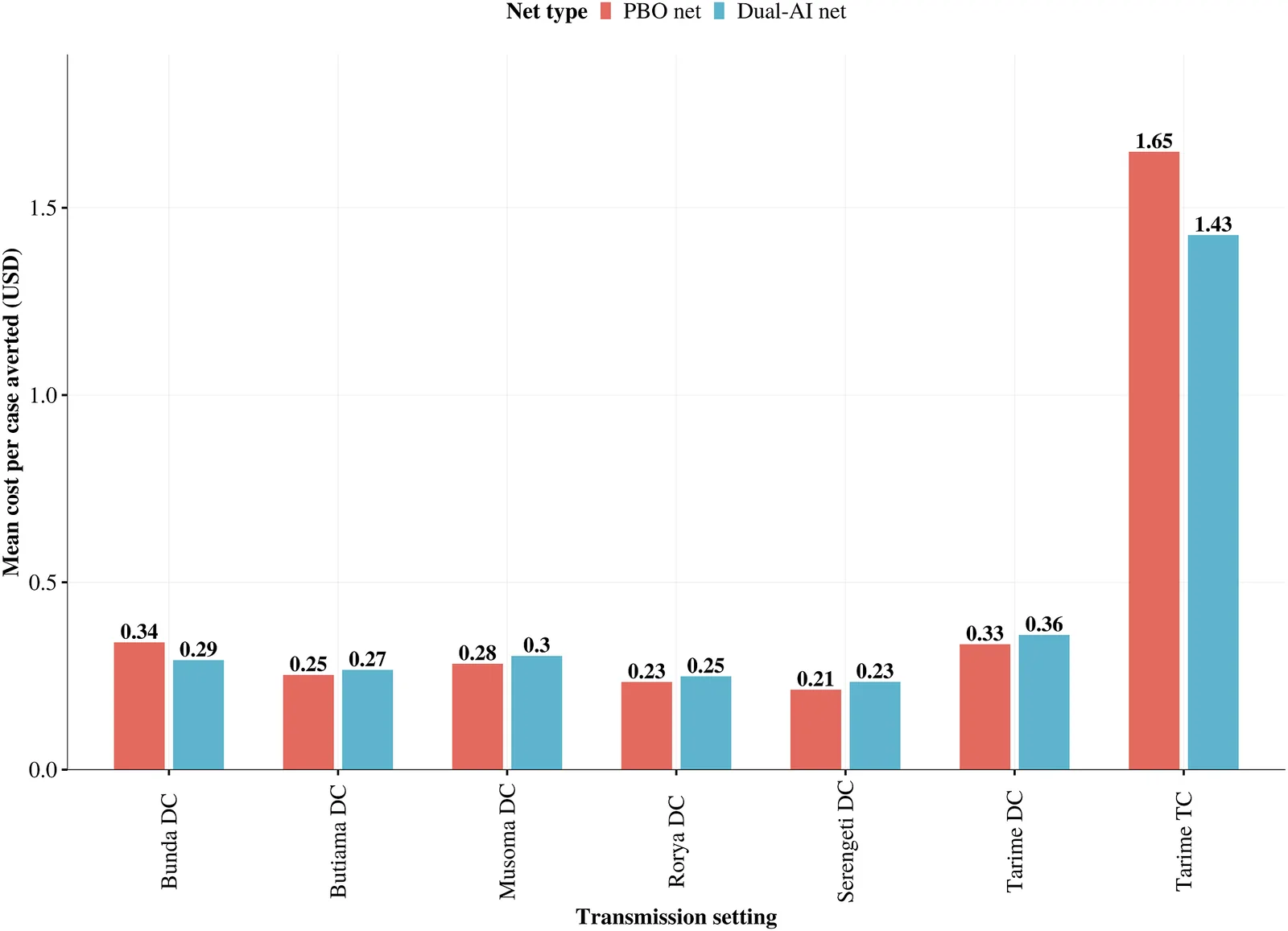
Mean cost per case averted (USD) for PBO and chlorfenapyr-α-cypermethrin (Dual-AI) nets across council-level transmission settings in Mara region over a three-year procurement cycle per 10,000 population. In most councils, both net types achieved comparable cost per case averted, with chlorfenapyr-α-cypermethrin nets remaining cost-effective despite higher procurement costs. Cases averted were estimated using OpenMalaria simulations at 80% net access relative to a pyrethroid-only counterfactual baseline. *Abbreviations*: DC, district council; TC, town council.

The cost-benefit analysis compared the cost-effectiveness of chlorfenapyr-α-cypermethrin and PBO nets relative to the pyrethroid-only baseline across transmission strata, expressed as cost per case averted (USD) over a three-year deployment cycle per 10,000 population. In Mara, the total three-year deployment costs were 17,493 USD for PY, 22,829 USD for PBO nets, and 27,411 USD for chlorfenapyr-α-cypermethrin nets across all three distribution channels combined. Despite the higher per-unit procurement price of chlorfenapyr-α-cypermethrin nets, the greater number of cases averted resulted in a comparable cost per case averted relative to PBO across all transmission settings (Fig. 10). In moderate transmission settings, which represent the majority of Mara councils, chlorfenapyr-α-cypermethrin nets averted cases at a mean cost of 0.25 USD per case (range: 0.18–0.39 USD), compared with 0.24 USD (range: 0.17–0.34 USD) for PBO nets. Across all transmission strata, chlorfenapyr-α-cypermethrin nets delivered equivalent or greater health impact over a total three-year deployment, supporting their cost-beneficial deployment particularly in moderate and high transmission councils (Fig. 10). The markedly higher cost per case averted in Tarime TC reflects the elevated transmission intensity in that setting, where a greater absolute burden limits the relative gains achievable within the 3-year cycle.

We further assessed the impact of coverage variation on the cost required per case averted (Supplementary Text S2). Interestingly, the impact varied between the high-transmission council (Tarime TC) and moderate-transmission councils. In high-transmission councils, we observed a higher cost per case averted at higher coverage levels than in moderate-transmission settings (Supplementary Fig. S2). This was probably because higher ITN coverage led to higher costs, and in a moderate-transmission setting, higher ITN coverage results in a greater reduction in malaria cases. However, in a high-transmission setting, increased coverage has only a limited impact on malaria incidence due to the very high exposure to infectious bites (Kamber et al., 2025).

## 4 Discussion

To inform the Tanzanian National Malaria Control Programs, we used an individual-based model of malaria transmission and epidemiology and assessed the epidemiological and economic impacts of three ITN types (dual-AI chlorfenapyr-α-cypermethrin, PBO, and standard pyrethroid-only nets, PY). Our findings indicate that the deployment of chlorfenapyr-α-cypermethrin nets at access levels consistent with WHO recommendations (≥ 80%) offers a better impact than PY or PBO ITN for achieving and sustaining downward transmission stratum transitions within a three-deployment cycle horizon.

The findings of this study demonstrate the significant effectiveness of the dual-active ingredient long-lasting nets, specifically the chlorfenapyr-α-cypermethrin nets, compared to the PBO and pyrethroid nets. The higher number of cases averted and lower incidence observed when the chlorfenapyr-α-cypermethrin nets are deployed through mass distribution support our hypothesis that maximizing insecticide exposure and net access enhances vector mortality, hence malaria control. The findings also demonstrate that the protective impact of chlorfenapyr-α-cypermethrin nets is sustained over a longer period compared to other net types. Based on parameterizations derived from field studies, which indicate slower decay in the killing and deterrence effects of these nets, the model projects higher malaria cases averted in the third year of distribution, particularly when compared to PBO nets. Beyond individual protection, ITNs exert a community-level effect through their impact on mosquito populations. As ITNs kill mosquitoes, the overall mosquito population declines, which in turn reduces transmission pressure across the entire community, including among individuals who do not use a net. This mass killing effect lowers the EIR by reducing both the density of infectious mosquitoes and the infectivity of humans to mosquitoes, 67.5% (95% CI: 47.6–79.6%) for the chlorfenapyr-α-cypermethrin nets and 56.4% (95% CI: 32.1–75.7%) for the PBO nets, as fewer people are infected and available to transmit the parasite. The resulting reduction in EIR amplifies the overall impact of ITN deployment beyond what would be expected from individual protection alone, and this effect is particularly pronounced in settings with lower transmission rates where even modest reductions in mosquito density can translate into a more substantial decline in malaria burden.

This study agrees with several studies done in Tanzania and across the globe in assessing the impact of the next-generation nets compared to other nets. The famous field study in Misungwi District, Tanzania, proved the greater durability of the chlorfenapyr-α-cypermethrin nets and their impact over the PY and PBO in a randomized-controlled trial (Matowo et al., 2023; Accrombessi et al., 2024). The findings of the Misungwi study showed the higher impacts of the chlorfenapyr-α-cypermethrin nets, causing a lower malaria transmission in the settings using this net type. There was even protection of non-users in the chlorfenapyr-α-cypermethrin nets arm compared to another arm that had high net access distribution of pyrethroids (Thickstun et al., 2025). Around sub-Saharan Africa, distributions of dual-active ingredient insecticide-treated nets have been done, portraying a reduction in malaria incidence in Burkina Faso (25%), northern Mozambique (75%), western Mozambique (26%), Nigeria, and Rwanda (13%) (PATH, 2023; Shannon et al., 2024).

Previous studies have highlighted the potential value of insecticide combinations in malaria vector control, particularly in settings affected by pyrethroid resistance (Tusting et al., 2015). In the present study, we parameterized the net using data from an experimental study in an area in Tanzania with mosquitoes resistant to PY (Champagne et al., 2025), which explains the poor impact of PY nets. The deployment of chlorfenapyr-α-cypermethrin nets resulted in greater reductions in malaria morbidity than deployment of PBO, and consistently outperformed pyrethroid-only strategies. These findings indicate that dual-active ingredient strategies can achieve higher effectiveness in reducing malaria burden under a range of transmission settings. Given the widespread challenges of pyrethroid resistance and declining durability of conventional ITNs across sub-Saharan Africa, the superior performance of chlorfenapyr-α-cypermethrin-containing strategies observed in this study supports their potential programmatic value. While our results do not directly address the evolution or spread of resistance, the higher effectiveness of chlorfenapyr-α-cypermethrin deployment, which has been observed together with longer durability in individual protection to the third year, suggests an added operational advantage. The dual-AI formulation may contribute to resistance management through the combination of insecticides. Such approaches align with WHO objectives (WHO, 2025a, 2025b) toward sustaining malaria control gains and progressing toward the 2030 malaria targets.

In many malaria-endemic countries, including Tanzania, ITN procurement and distribution through multiple delivery channels are largely supported by external funding from international donors such as the Global Fund and USAID (President’s Malaria Initiative, 2023). Recent reductions in global health funding highlight the importance of optimizing the allocation of available resources. Our findings provide a strong epidemiological rationale for this resource prioritization; although dual-active ingredient nets are associated with higher unit costs, the superior impact demonstrated in this model combined with evidence of sustained efficacy through the third year of distribution suggests that investment in chlorfenapyr-α-cypermethrin nets can deliver greater cases averted per resource unit spent compared to conventional ITNs. The longer-lasting protection profile of chlorfenapyr-α-cypermethrin nets (demonstrated by minimal resistance development and maintained deterrence through Year 3) means that programmes may redirect toward complementary interventions after a certain number of deployments while maintaining malaria control momentum. Furthermore, non-users would also benefit from the community protection by these chlorfenapyr-α-cypermethrin nets.

Despite the promising results, this study has several limitations that warrant consideration. Experimental hut trials (Martin et al., 2024; Champagne et al., 2025) and parameterizations used to inform the model, while derived from controlled settings, may not fully capture real-world conditions, where environmental heterogeneity, human behavior, mosquito species composition, and levels of insecticide resistance can influence net performance (Ranson and Lissenden, 2016). In this analysis, a single parameterization was applied for each ITN type, and therefore potential variation in effectiveness arising from differences in vector species or resistance intensity was not explicitly represented. The net efficacy parameters reflect the impact of ITN on vector population with a resistance profile of the Tanzania site where the entomological study took place. The net efficacy parameters were held constant over the projection period and applied uniformly across councils. In reality, pyrethroid resistance in Tanzania likely varies substantially by location and would probably increase in prevalence and intensity over time (Tungu et al., 2023). Future research should assess the long-term effectiveness of chlorfenapyr-α-cypermethrin nets across diverse ecological settings and mosquito populations, accounting for heterogeneity in vector species composition and resistance profiles. Finally, note that our study compared the impact of the three ITN types across different channels, and it was not designed to compare the impact of different channels within themselves. Different channels would have different impacts across different age groups while we look at total cases averted in the whole population; thus, distribution channels that disproportionately protect younger children may confer greater health benefit, especially against severe malaria cases, than reflected by total cases averted alone, as reported.

## 5 Conclusions

Dual-active ingredient nets represent a significant advancement in malaria vector control. Our findings demonstrate that chlorfenapyr-α-cypermethrin nets deliver superior and sustained protection over longer deployment periods, reducing malaria incidence while slowing resistance development through their combination of mechanistically distinct insecticides. Validated by field evidence from Tanzania and sub-Saharan Africa, deploying these nets with adequate population coverage (≥ 80%) would have the greatest impact on clinical malaria cases compared to standard pyrethroid or PBO-based nets. However, the effectiveness of these interventions is contingent on programme coverage, as net access levels vary across distribution cycles. The proportion of individuals benefiting from enhanced ITNs at each cycle directly determines the resulting impact on clinical and severe malaria cases. These results suggest that chlorfenapyr-α-cypermethrin ITNs could contribute significantly to reducing malaria morbidity in diverse settings, offering a promising and operationally justified strategy for malaria control programmes to sustain transmission reductions toward 2030 elimination targets. This reinforces the importance of comprehensive vector control strategies that combine novel insecticides with high population coverage in malaria elimination efforts.

## Ethical approval

Not applicable.

## CRediT authorship contribution statement

**Gloria Salome Shirima:** Conceptualization, Methodology, Software, Writing - original draft, Writing - review & editing. **Mponeja Gitanya:** Conceptualization, Data curation Validation, Writing - review & editing. **Charles D. Mwalimu:** Conceptualization, Data curation Validation, Writing - review & editing. **Nakul Chitnis:** Conceptualization, Investigation, Methodology, Writing - review & editing, Supervision. **Silas Mirau:** Conceptualization, Methodology, Writing - review & editing, Supervision. **Samson Kiware:** Conceptualization, Investigation, Methodology, Data curation, Validation, Writing - review & editing, Supervision. **Thiery Masserey:** Methodology, Data curation, Validation, Visualization, Writing - review & editing, Supervision.

## Statement on the use of AI-assisted technologies

While preparing this article, the authors used OpenAI’s to correct grammatical errors and improve readability. After using this tool, the authors reviewed and edited the content as needed. The authors take full responsibility for the content of the published article.

## Funding

This work was supported, in part, by the Gates Foundation [INV-070227, INV-025569] and the Swiss federal government’s Department of Education and Research. Under the grant conditions of the Foundation, a Creative Commons Attribution 4.0 Generic License has already been assigned to the Author Accepted Manuscript version that might arise from this submission.

## Declaration of competing interests

The authors declare that they have no known competing financial interests or personal relationships that could have appeared to influence the work reported in this paper.

## Data Availability

The data supporting the conclusions of this article are included within the article and its supplementary files. All the codes used are publicly available on GitHub and archived on Zenodo (https://doi.org/10.5281/zenodo.20209778).
