# Supplementary material for: Comparative malaria impact of chlorfenapyr-α-cypermethrin nets versus pyrethroid-only and pyrethroid-piperonyl butoxide nets across high and moderate transmission settings in Tanzania: A modelling study
Source: Curr Res Parasitol Vector Borne Dis. 2026 Aug 25;10:100429. doi: 10.1016/j.crpvbd.2026.100429 (PMC13553524; doi:10.1016/j.crpvbd.2026.100429)
Supplement: Supplementary file 1 [file mmc1.pdf]

Supplementary Material

Supplementary Text S1: Conversion of Prevalence to EIR

To parameterize transmission intensity in our model, we required estimates of the entomological inoculation rate (EIR) corresponding to observed parasite prevalence (PfPR) in individuals aged 5-16 years. Because direct EIR measurements were not available, we derived EIR from prevalence using a simulation-based mapping informed by OpenMalaria. This approach follows the methodology described in the study "Distribution of malaria exposure in endemic countries in Africa considering country levels of effective treatment", which uses OpenMalaria simulations to establish a relationship between prevalence and EIR that accounts for treatment coverage. The relationship between prevalence and transmission intensity is nonlinear and depends on treatment coverage. We therefore adopted a Hill function to describe this relationship. The Hill function expresses prevalence as a function of EIR and treatment level:

$$p(x, E) = [p\_max(E) * x^{n(E)}] / [K(E)^{n(E)} + x^{n(E)}]$$

where p is prevalence, x is EIR, and E is treatment coverage. The parameters p_max(E), K(E), and n(E) represent the maximum achievable prevalence, the half-saturation EIR, and the shape of the curve, respectively.

Estimation procedure: Simulation outputs were generated using OpenMalaria across a range of EIR values and treatment levels. For each treatment level, a Hill function was fitted to describe the relationship between prevalence and EIR, yielding estimates of K, n, and p_max for each level. The dependence of the Hill parameters on treatment was modeled using functional forms consistent with previous work. Specifically:

$$K(E) = K1 * \exp(K2 * E)$$
$$n(E) = n1 * E^2 + n2 * E + n3$$
$$p\_max(E) = p1 * \exp(p2 * E)$$

These forms were selected based on their ability to adequately capture variation in the parameters across treatment levels while maintaining simplicity. To estimate EIR from observed prevalence, the Hill function was analytically inverted:

$$x = K(E) * [p / (p\_max(E) - p)]^{(1 / n(E))}$$

This equation allows calculation of EIR given prevalence and treatment coverage. Observed prevalence values per council were converted to proportions by dividing by 100. The inverse Hill function was then applied to estimate EIR for each observation using the corresponding treatment level. The method assumes that prevalence values lie strictly between zero and the maximum achievable prevalence p_max(E). The fitted parameter functions are continuous in treatment coverage, allowing estimation of EIR for intermediate values not explicitly included in the simulation outputs. This enables application of the method across a range of realistic treatment scenarios.

Fitted prevalence-EIR relationships

The fitted Hill curves reproduce the expected sigmoidal relationship between EIR and prevalence across treatment levels, with clear saturation at higher transmission intensities and downward shifts with increasing treatment coverage. The figure below illustrates these fitted relationships across treatment levels.

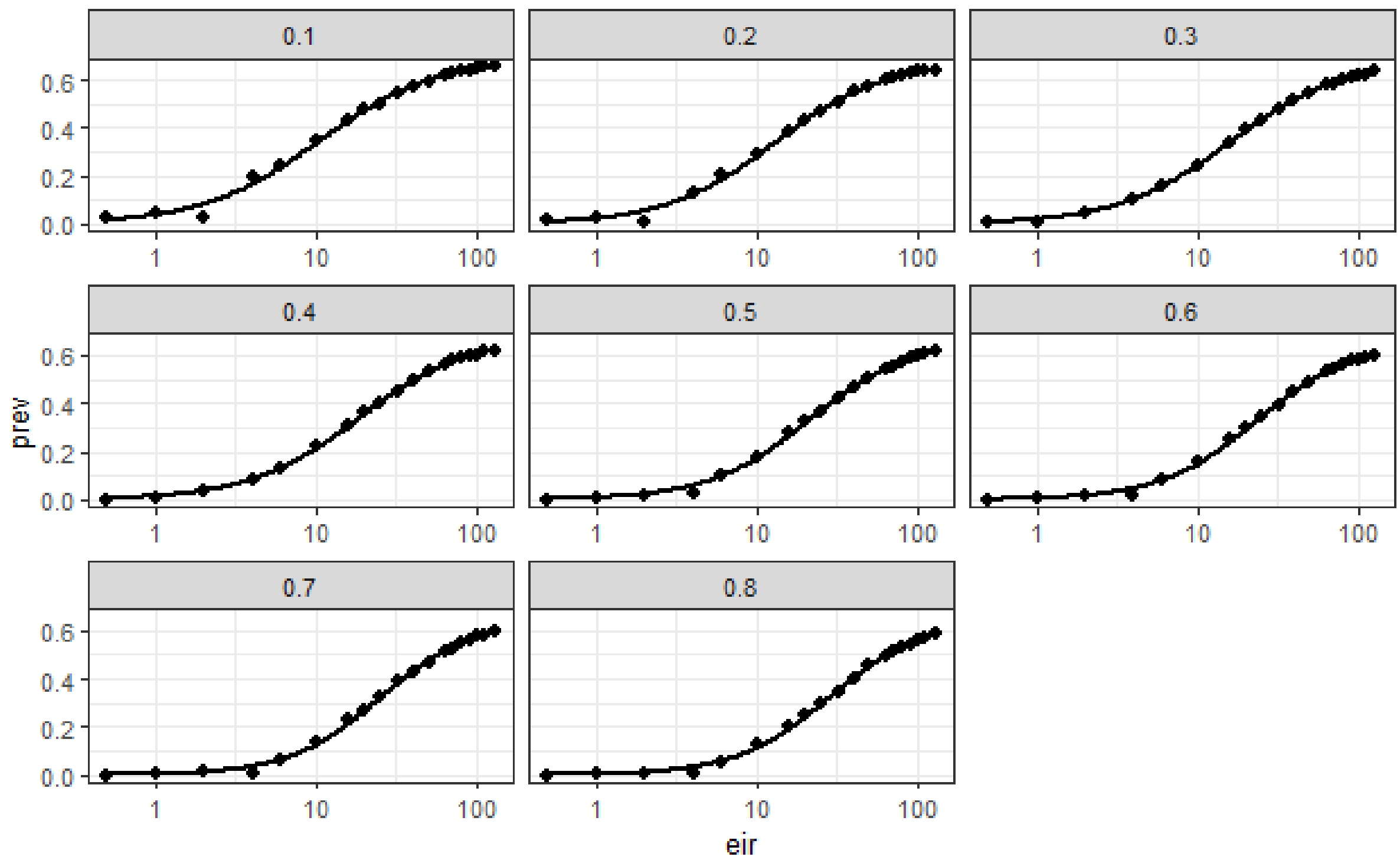

Supplementary Figure S1: Relationship between prevalence and EIR on varying level of treatment

Supplementary Text S2: Cost per case averted on varying coverage

To assess how cost-effectiveness varies with intervention scale-up, we calculated the mean cost per case averted for the Dual-AI and PBO nets, relative to pyrethroid-only nets, across varying ITN coverage levels in each council (Figure S2). In most councils, cost per case averted decreased as coverage increased, reflecting greater efficiency at higher scale-up. In Tarime TC, however, cost per case averted increased markedly with coverage, consistent with heterogeneity in mosquito exposure limiting further reductions in incidence in high-transmission settings despite increased investment (Kamber et al., 2025)

At 30%:

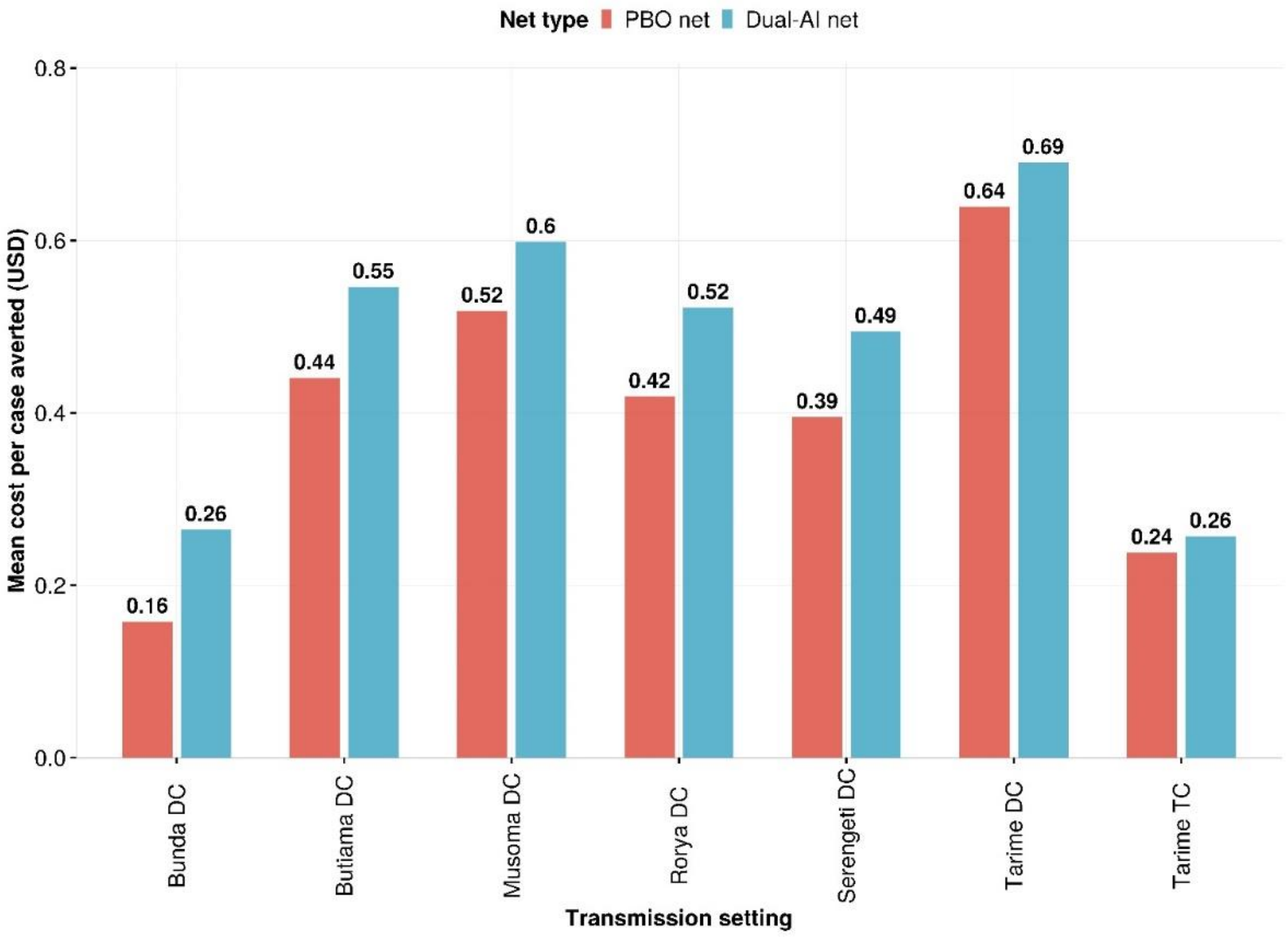

Net type PBO net Dual-AI net
Mean cost per case averted (USD)
0.8
0.6
0.4
0.2
0.0
0.16
0.26
0.44
0.55
0.52
0.6
0.42
0.52
0.39
0.49
0.64
0.69
0.24
0.26
Bunda DC
Butiama DC
Musoma DC
Rorya DC
Serengeti DC
Tarime DC
Tarime TC
Transmission setting

At 40%:

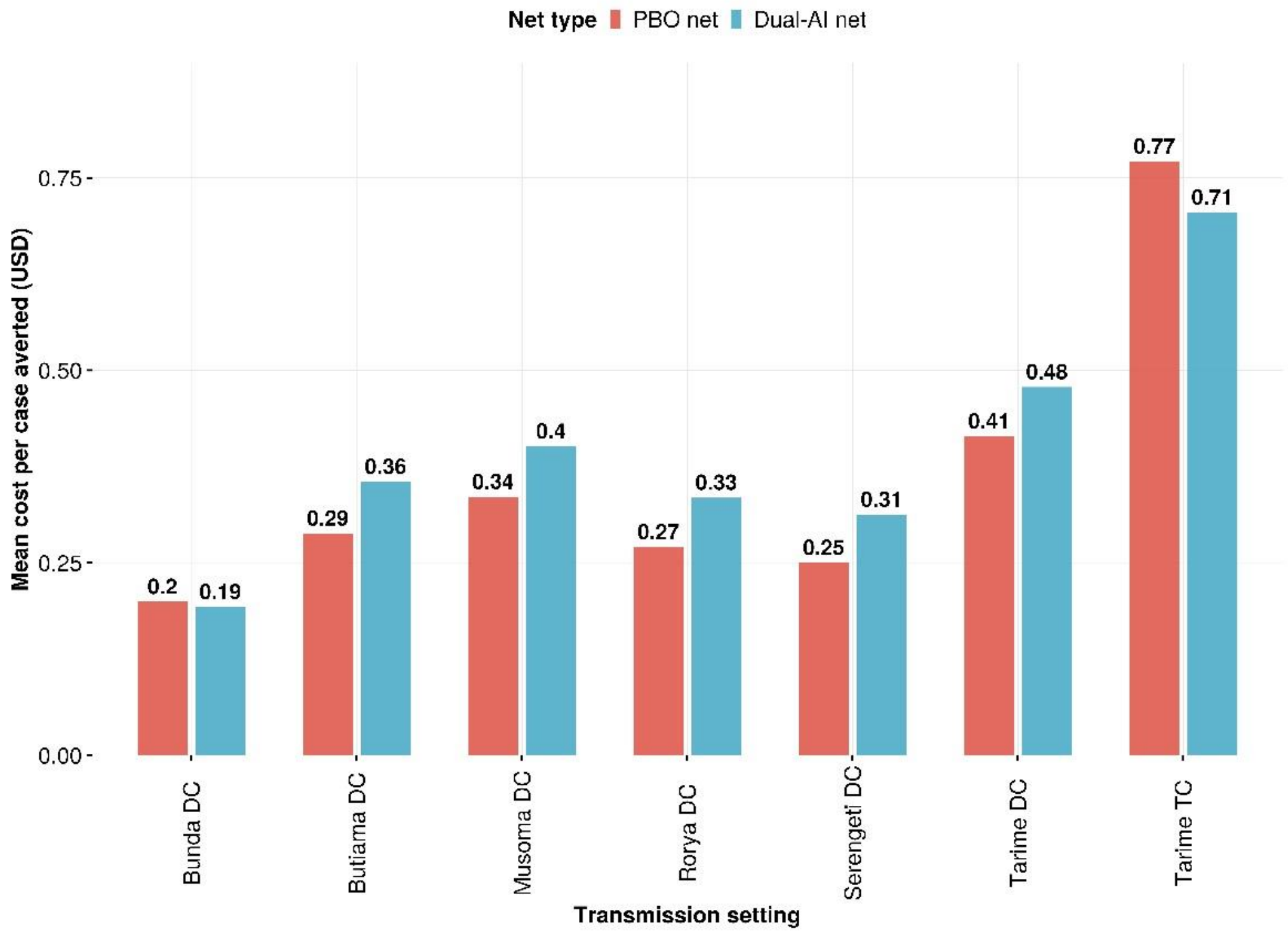

Net type PBO net Dual-AI net
Mean cost per case averted (USD)
0.75
0.50
0.25
0.00
0.2
0.19
0.29
0.36
0.34
0.4
0.27
0.33
0.25
0.31
0.41
0.48
0.77
0.71
Bunda DC
Butiama DC
Musoma DC
Rorya DC
Serengeti DC
Tarime DC
Tarime TC
Transmission setting

At 60%:

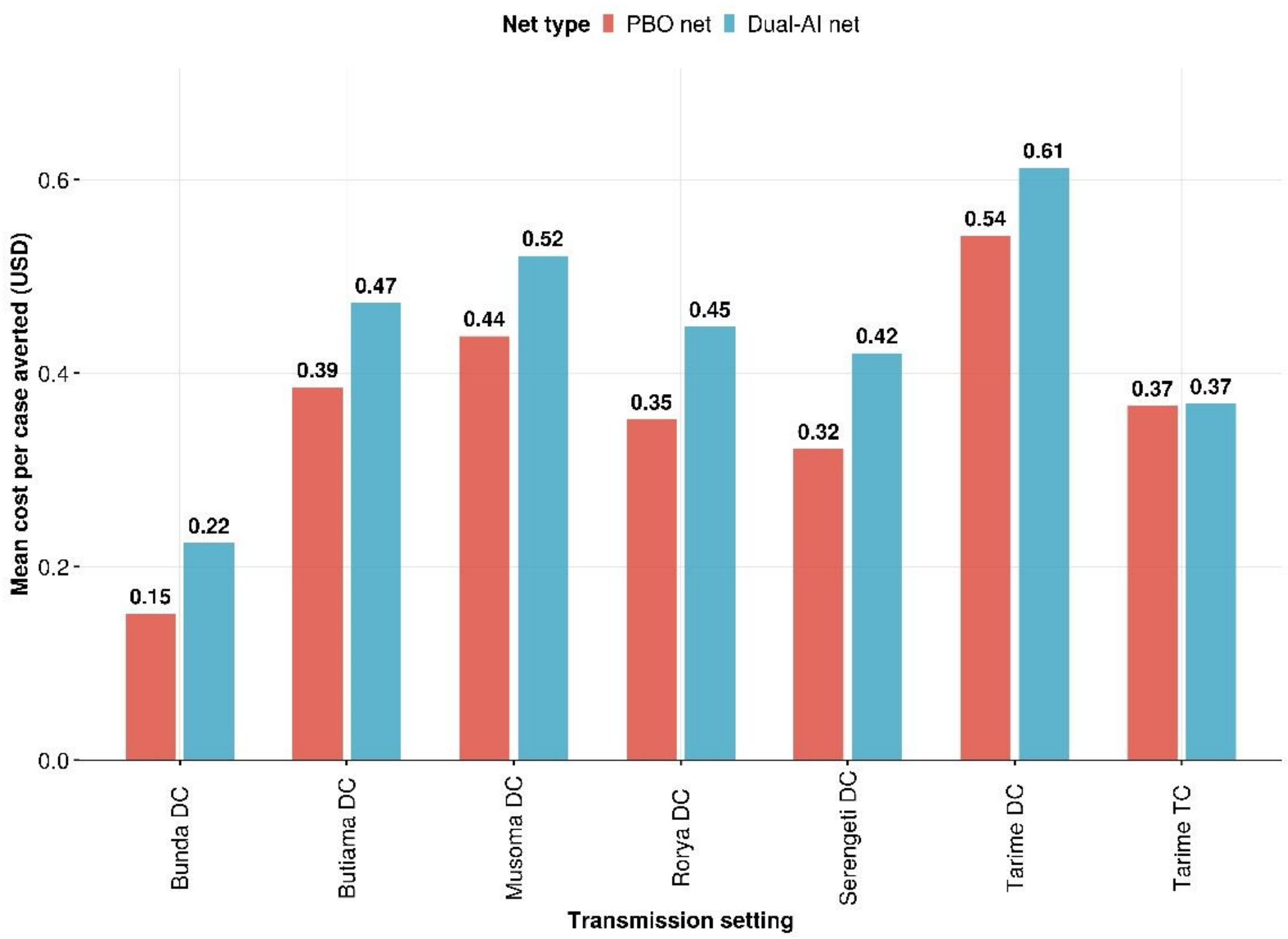

At 80%:

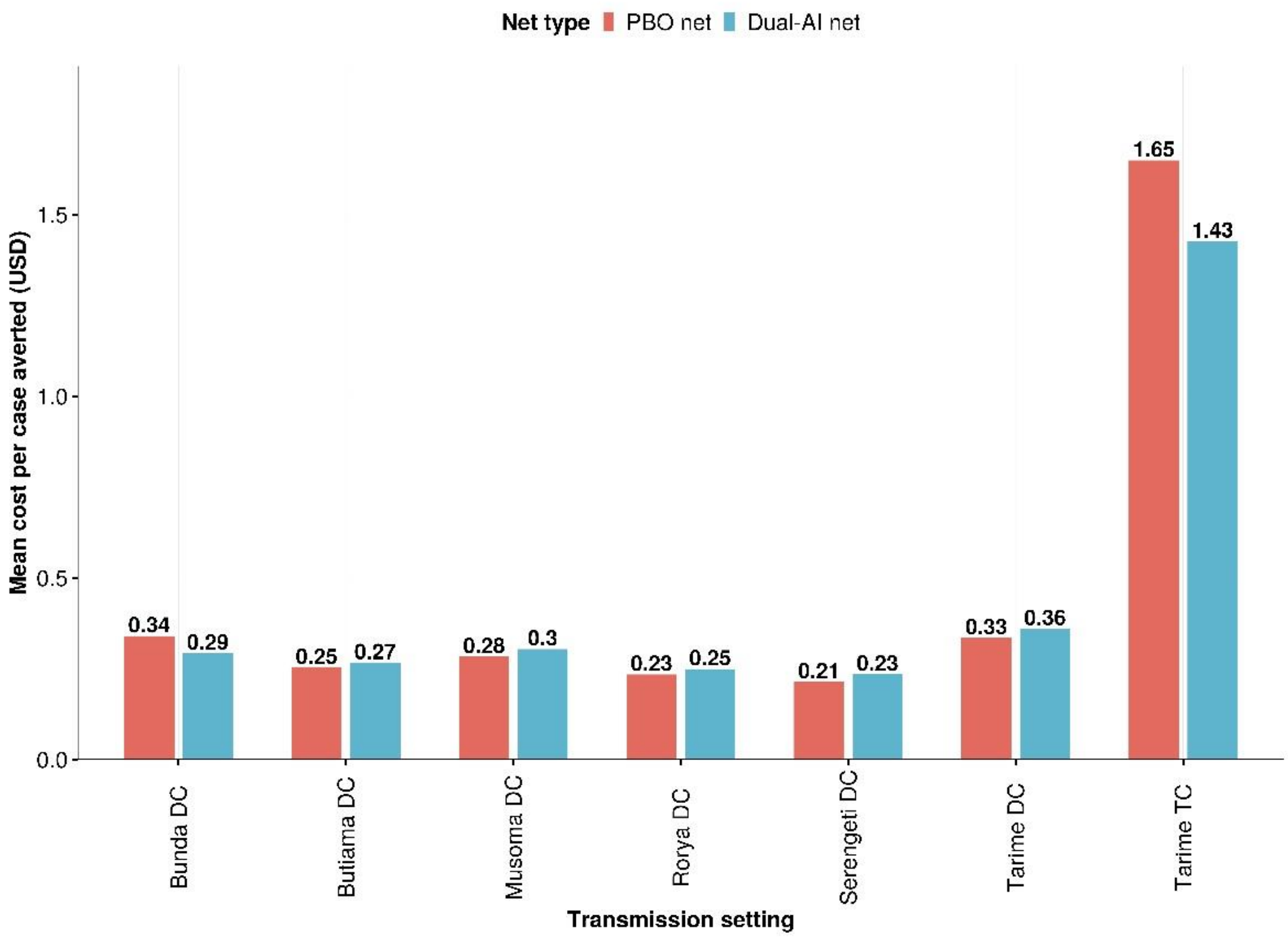

Supplementary Figure S2. Mean cost per case averted (USD) for the Dual-AI and PBO nets, relative to pyrethroid-only nets, across varying ITN coverage levels in seven councils. Cost per case averted generally decreased with increasing coverage, except in Tarime TC, where it increased markedly at higher coverage.

Supplementary Table S1. Deterrence and mosquito-killing effects of ITNs used in the simulations. Parameters describe the protective effect of each net type against pyrethroid-resistant Anopheles mosquitoes. Deterrancy (x) represents the reduction in host availability to mosquitoes, such that host availability becomes (1 − x) × pre-intervention availability. Pre-prandial killing effect (y) represents the increase in mosquito mortality before feeding, such that the probability of successfully biting a protected host becomes (1 − y) × pre-intervention probability. Post-prandial killing effect (z) represents the increase in mosquito mortality after feeding, such that the probability of a mosquito escaping after feeding on a protected host becomes (1 − z) × pre-intervention probability.

Net type*	Deterrency	Pre-prandial killing effect	Post-prandial killing effect
Pyrethroid net	0.37	0.08	0.05
PBO nets	0.7	0.47	0.44
Chlorfenapyr-α-cypermethrin nets	0.19	0.41	0.42

*Values are unitless, expressed as proportions per feeding attempt.

Supplementary Table S2. Weibull distribution parameters for ITN vector effects used in the model.

Net type	Deterrency λ	Deterrency k	Pre-prandial killing λ	Pre-prandial killing k	Post-prandial killing λ	Post-prandial killing k
Pyrethroid net	2.3	1.6	2.3	1.6	2.3	1.6
PBO net	1.6	1.8	1.3	1.7	1.1	1.7
Chlorfenapyr-α-cypermethrin nets	2.4	2.4	1.3	1.8	1.8	1.9

λ denotes the Weibull scale parameter and k the shape parameter. All parameters are dimensionless.
